# Expanding the Ingredient Basket in Aquaculture: Growth Performance and Feed Utilization of Australian Hybrid Abalone (*Haliotis laevigata × H. rubra*) Fed Methanotrophic Single Cell Protein

**DOI:** 10.1155/anu/7291857

**Published:** 2025-03-03

**Authors:** Michael J. Salini, Weifang Wang, Thomas S. Mock, Muhammad A. B. Siddik, Matthew K. Jago, Kelsey L. F. Bews, David S. Francis

**Affiliations:** ^1^Nutrition and Seafood Laboratory (NuSea.Lab), School of Life and Environmental Sciences, Deakin University, Queenscliff, Victoria, Australia; ^2^State Key Laboratory of Mariculture Biobreeding and Sustainable Goods, Yellow Sea Fisheries Research Institute, Chinese Academy of Fishery Sciences, Qingdao 266071, Shandong, China

**Keywords:** Australian hybrid abalone, methanotrophic bacteria, nutrition, protein hydrolysate, single cell protein, soy protein concentrate

## Abstract

There is growing interest in the use of single cell proteins (SCPs) derived from methanotrophic bacteria for inclusion in aquafeed to reduce reliance on other, potentially less sustainable proteins. This two-part experiment aimed to investigate first (i) the dose effect of replacing soy protein concentrate with SCP in Australian hybrid abalone diets (*Haliotis laevigata Haliotis rubra*) and second, (ii) the potential for improved palatability of the SCP by using commercially produced liquid protein hydrolysate (PH). This was assessed in a 2 × 2 factorial experimental design. The diets were formulated to be iso-proteic (~40% crude protein as fed) and iso-energetic (~18 MJ kg^−1^ as fed). The diets (SCP 0%, SCP 5%, SCP 10%, SCP 20%, SCP 0% + PH, and SCP 20% + PH) were fed to the abalone (~9.25 g initial weight) once daily in the evening for 94 days to apparent satiation. The growth performance and feed utilization of abalone fed with up to 10% SCP was comparable to the control; however, there was a significant reduction in most parameters at the highest inclusion of 20%. In the second experiment, there was a decline in measured protein and energy retention in the abalone fed 20% SCP, with a concomitantly lower apparent biological value. Positively, there were significant improvements in the apparent digestibility of the diet at the 20% SCP inclusion. There was no significant impact of using the PH on growth performance or feed intake. No effects were observed on the digestive enzyme activity (*α*-amylase, lipase, and trypsin) examined among the treatments. Considering these results collectively, the maximum recommended inclusion of methanotrophic SCP meal should be 10% for hybrid abalone. Reasons for the decline in performance at the highest inclusion may be related to palatability; however, this remains to be fully confirmed.

## 1. Introduction

Recently, an increasing number of nutritional studies on Australian hybrid abalone (*Haliotis laevigata × Haliotis rubra*) have expanded our knowledge of their specific protein requirements and interactions with temperature [1–4]. The hybrid of *H. rubra* and *H. laevigata* is successfully produced under Australian growing conditions owing to gains from heterosis, in particular relating to fast growth [5]. However, one aspect that has not received much attention is the raw material basket of ingredients used to formulate their diet [6, 7]. During the early stages of development, abalone diets consist of microalgae, and then as early juveniles in the wild, this switches to a macroalgae diet for the remainder of their life. Therefore, when it comes to formulated feed for abalone, plant-based diet solutions are common in the published literature [6]. The use of commonly available and typically low-cost ingredients such as wheat, canola, soy, and other grains are used in the production of abalone feeds globally, with additional algal based supplements common [6, 8].

The use of novel protein sources such as single cell proteins (SCPs) is not a new concept in aquaculture nutrition. Recent research on the use of SCP has attracted considerable attention in the literature due to their favorable chemical composition (e.g., high protein concentration), scaled-up production facilities, formulation flexibility, and sustainability credentials [9, 10]. Encouragingly, the emerging industries invested in the SCP production technology are poised to capture the demand from feed producers aiming to reduce their environmental footprint. Accordingly, there is considerable scope to use SCP in the diet of Australian hybrid abalone, which has a higher protein demand compared to other abalone species such as the greenlip abalone (*H. laevigata*) [11]. Traditionally, the choices for protein sources in abalone diets are constrained in a commercial sense as there is a reluctance to utilize certain plant or land animal proteins due to cost, availability, or even impacts on the flavor profile of the abalone meat [6, 8]. Moreover, the fishmeal inclusion is typically low (≤10%) [12].

Across a range of aquatic species, promising results using SCP have been reported with a general observation that at lower inclusion levels animals respond similarly to control diets containing no SCP. However, at higher SCP inclusion levels, there can be negative impacts on production performance. For example, in their study on *Haliotis discus hannai*, the authors reported optimal performance at the 25% fishmeal replacement inclusion of *Clostridium autoethanogenum* bacterial protein replacing fishmeal. However, there was a significant decline for several measured parameters beyond 50% inclusion level [13]. Further to this, Delamare-Deboutteville et al. [14] found that in juvenile Asian seabass (*Lates calcarifer*), up to 66% of the included fishmeal could be substituted with mixed phototrophic bacterial biomass; however, beyond this, both growth and feed conversion ratio (FCR) were significantly negatively affected. Similar results with rainbow trout (*Oncorhynchus mykiss*) are also reported with reduced feed intake and hence growth ascribed to a palatability effect of *Methylococcus capsulatus* [15].

Negative effects on feed intake may be caused by the inclusion of SCP products as demonstrated in past studies. Pilmer et al. [16] attempted to mask a reduced palatability effect using protein hydrolysate (PH) and garlic powder in diets for yellowtail kingfish (*Seriola lalandi*). The authors found that despite the addition of these attractants, there was still a reduction in feed intake at higher inclusions of SCP, leading to reduced weight gain. Positively, they found that the reduction in weight gain was likely caused by reduced feed intake alone and, therefore, overcoming this could allow for higher SCP supplementation. Similarly, graded levels of SCP were fed to Japanese yellowtail (*Seriola quinqueradiata*) and again reduced feed intake led to reduced growth performance [17]. Unlike in fish, however, the natural feeding behavior of abalone tends to be “slow grazing” and studies examining diet attractiveness or palatability in abalone are scarce. Therefore, understanding and improving the feed intake response of Australian hybrid abalone is of considerable interest. As an example, the weight gain of *H. midae* was improved by the addition of high-quality PH products, highlighting the potential to improve intake in a haliotid species [18].

As such, in a two-part experiment, the primary objective of the present study was to test the dose–response effect of a methanotrophic SCP while replacing soy protein concentrate in diets fed to Australian hybrid abalone. In the second experiment, we hypothesized that feed intake may be impacted by a high-level inclusion of SCP. Therefore, we aimed to improve the feed intake response of Australian hybrid abalone with a PH supplementation in a 2 × 2 factorial approach.

## 2. Materials and Methods

### 2.1. Animal Husbandry and Setup

No animal ethics approval was required for the experiment; however, any interventions were performed according to the guidelines detailed in the Australian Code for the Care and Use of Animals for Scientific Purposes (8th edition) [19]. Australian hybrid abalone (*H. laevigata × H. rubra*), each weighing approximately 9 g, from a graded stock population, were sourced from Jade Tiger Abalone, Indented Head, Victoria, for both experiments. The abalone were picked and held in a clean water system for approximately 1 week for acclimatization to the system conditions. During this time, the abalone were fed a commercially relevant diet (manufactured to the same specification as the control diet used for the experiment and described below). Both experiments were conducted in the same experimental system following similar procedures as previously reported, at the Deakin University Queenscliff Marine Science Centre, Queenscliff, Victoria, Australia [2].

The experimental system consisted of 18 × 12 L blue plastic aquaria (385 mm × 286 mm × 105 mm internal dimensions), fitted with hides constructed with tiles, similar to those used previously [3]. The system was supplied with flow through, temperature-controlled seawater (Aquahort Ltd., Australia), at approximately 0.5 L min^−1^ to each tank. The average water temperature during the experimental period was 19.9 ± 0.39°C (Hobo, Australia). The photoperiod was set to constant very low light during experimentation. Each aquarium was supplied with constant aeration and oxygen levels were monitored daily (>90% saturation) using an OxyGuard Handy Polaris (Technolab, Australia).

### 2.2. Ingredients, Diet Production, and Nutrient Retention Test

The analyzed composition of the key test ingredients including methanotrophic SCP PRO-DG, supplied by String Bio Pvt. Ltd., Bangalore, India, and soy protein concentrate are presented in Table 1. The experimental diets were formulated to meet or exceed the known nutritional requirements for Australian hybrid abalone. Where nutrient requirements were not available, they were estimated based on other closely related haliotid species and other relevant commercial considerations. All dry raw materials were milled to d90 <800 µm using a Perten Lab Mill 3100 (Perkin Elmer, Victoria, Australia) prior to use. The mash ingredients were thoroughly mixed in an upright Hobart mixer (Hobart, Australia), before oil and water (approx. 30%) were added to form a stiff dough. The six iso-proteic and iso-energetic disk-shaped diets (approx. ~4 mm diameter) were produced using a bench top pasta machine (Dolly, La Monfarina, Winequip, Victoria, Australia). The formulation of the diets for both experiments is presented in Table 2. In the first experiment, the control (diet SCP 0%) was formulated with soy protein concentrate as the main protein source. SCP was included at 5%, 10%, and 20% (diets SCP 5%, SCP 10%, and SCP 20%) in subsequent diets, replacing protein from soy protein concentrate. In experiment 2, the SCP 0% and SCP 20% diets from experiment 1 were used and an additional two diets were produced in the same way, with commercially produced liquid PH added on top of the formulation. The experimental diets were oven dried overnight to constant moisture, at 45°C. The diets were packed into suitable containers and stored in a cool dry place prior to use. The formulation and analyzed composition of each experimental diet are presented in Table 2.

The diets were subjected to a retention test whereby a representative sample of approximately 5 g was soaked prior to collection and drying following the procedure described below. The system (as described above) was supplied with flow through, temperature-controlled seawater (~20°C), at approximately 0.5 L min^−1^ and each aquarium was supplied with constant aeration. The diets were randomly allocated to the system with each having three replicate tanks. The pellets were placed into the tanks and left to soak for 16 h and then the pellets were physically removed by siphoning and collection in a mesh screen, then dried to constant weight. The results are presented on a dry matter basis. The recovery (leaching loss) of each diet is presented in Table 3.

### 2.3. Experimental Procedures and Sampling

Prior to removal from the holding system, the abalone were relaxed with a small amount of magnesium sulfate (approximately 1 g L^−1^) to aid in removal. The abalone were picked in small batches (<10 animals) and then individually weighed and measured before allocation to their respective tanks. Twenty-four abalones weighing 9.26 ± 0.16 g with a shell length of 41.45 mm ± 0.35 mm were stocked into each of the aquaria. The experimental diets were randomly allocated to three replicate tanks and fed 6 days per week, once daily at 1600 h, during a 94-day period. Abalone were fed a preweighed, satiated ration, based on the response of the previous feeding day. Uneaten pellets were accounted for each day at ~0900 h by accurately counting the remaining pellets and then multiplying by the average weight of a feed pellet on an as-fed basis. If there were no pellets remaining the following morning, the feed rate was increased. If pellets were partly consumed, then a reasonable estimate was made on the remaining particles. This method was used to estimate feed intake in each tank following [2, 20]. The tanks were thoroughly cleaned following counting each day, with any uneaten pellets and faeces removed by siphoning to waste. Immediately prior to the commencement of the experimental allocation, six abalone of approximately average size from the source stock were stored at −20°C pending compositional analysis. Prior to final sampling, feeding was withheld for a 24-h period.

Upon termination of the growth experiment, the weight and length of each individual abalone were measured to calculate growth performance and feed efficiency indices. Three abalone from each tank were shucked and the soft tissue was weighed to calculate the relative proportion of soft tissue to shell mass composition. The soft tissue of the three abalone was then pooled and frozen pending compositional analysis.

### 2.4. Faeces Collection for Apparent Digestibility (AD) Assessment

During the last 2 weeks of the growth experiment, faeces were collected from each tank. The procedure involved siphoning the faeces at 1400 h each day, into a mesh screen, then rinsing with clean water into 15 mL conical bottom tube, before centrifuging and pouring off the remaining water prior to freezing the sample. The procedure was repeated daily until sufficient sample was obtained. The sample was then freeze dried and stored in a freezer until further analysis.

### 2.5. Sample Preparation and Chemical Analyses

All chemical analyses were conducted following standard or modified procedures [21]. The test ingredients and diets were homogenized prior to analysis. The frozen tissue samples were freeze dried to constant weight and then homogenized prior to analysis. The moisture was determined gravimetrically after oven drying at 105°C overnight. The ash content was determined gravimetrically after combustion in a muffle furnace at 550°C overnight. The nitrogen content was measured by Kjeldahl method using a Foss Kjeltech 2300 auto titrator (Foss, Sweden) and crude protein calculated (*N* × 6.25). The total lipid content was measured using a modified cold extraction following [22], using 2:1 dichloromethane: methanol.

The amino acid profile of the test ingredients and diets was determined by reverse phase high-pressure liquid chromatography (Infinity 1260, Agilent Technologies, USA) and presented in Tables 1 and 2. Briefly, the samples were hydrolyzed for 22 h in 6M hydrochloric acid at 110°C, filtered and diluted followed by precolumn derivatization using ophthaldialdehyde and fuorenylmethyloxycarbonyl chloride chemistry [23]. A Zorbax Eclipse Plus RP C18 column (3.5 µ, 150 mm × 0.46 mm) (Agilent Technologies, Santa Clara, USA) was used for separation and a diode-array detector was set to 338 and 262 nm for the detection of peaks (the latter for detection of the sarcosine standard and proline only). Peaks were identified by comparing to external standards while amino acids were quantified using internal standards of norvaline and sarcosine.

The yttrium oxide content of diet and faecal samples was determined after digestion in nitric acid and analysis by inductivity-coupled plasma mass-spectroscopy at the Plant Chemistry Research Laboratory of the School of Life and Environmental Sciences, Deakin University, Burwood Campus, VIC, Australia.

### 2.6. Digestive Enzyme Analysis

The digestive gland from three additional abalone from each tank was dissected for analysis. Prior to extraction in ultra-pure water (Milli-Q, Merck KGaA, Darmstadt, Germany), the samples (representing three individual abalone) from each replicate tank were pooled to create a unique sample for each tank replicate. The homogenate was centrifuged at 12,600 RCF for 25 min at 4°C. The supernatant was removed and stored on ice prior to analysis. Solubilized protein assay was performed on each sample prior to enzyme analysis, and the results of enzymatic activity were expressed as the activity per unit of tissue protein. All assays were performed using a microplate reader (FLUOstar Omega, BMG Labtech, Melbourne, Australia).

The protein assay kit II (#5000002, Bio-Rad Laboratories, NSW, Australia), using bovine serum albumin standard, was purchased for the experiment. The analysis was based on the Bradford [24] method of determining solubilized protein using an acidic dye-binding assay. Aliquots of each sample were mixed and prepared according to the manufacturer's instructions and added to a 96-well plate and absorbance was read in endpoint mode, with a discrete wavelength set to 595 nm at ambient temperature.

The *α*-amylase assay kit (#ab102523, Abcam, Australia), using nitrophenol standard, detects the activity of *α*-amylase through a two-step reaction. This is where *α*-amylase will cleave the substrate ethylidene-pNP-G7 to produce smaller fragments that are eventually modified by *α*-glucosidase, causing the release of a chromophore. Aliquots of each sample were mixed and prepared according to the manufacture instructions and added to a 96-well plate, and the absorbance was measured in slow kinetics plate mode, performing 20 cycles with a cycle time of 120 s and a discrete wavelength set to 595 nm at 25°C. Time points in the linear range (T1 = 2 min and T2 = 8 min) were chosen for the calculation of *α*-amylase activity.

The trypsin activity assay kit (#ab102531, Abcam, Australia), using *p*-nitroaniline (*p*-NA) standard, generates *p*-NA by cleaving a substrate with trypsin. Aliquots of each sample were mixed and prepared according to the manufacturer instructions and added to a 96-well plate, and the absorbance was measured in slow kinetics plate mode, performing 30 cycles with a cycle time of 120 s and a discrete wavelength of 495 nm at 25°C. Time points in the linear range (T1 = 10 min and T2 = 60 min) were chosen for the calculation of trypsin activity.

The lipase activity assay kit (#ab102524, Abcam, Australia), using glycerol standard, uses an OxiRed probe to monitor changes in the probe absorbance after hydrolysis with a triglyceride substrate that forms glycerol. Aliquots of each sample were mixed and prepared according to the manufacturer instructions and added to a 96-well plate, and the absorbance was measured in slow kinetics plate mode, performing 45 cycles with a cycle time of 120 s and a discrete wavelength of 570 nm at 37°C. Time points in the linear range (T1 = 20 min and T2 = 60 min) were chosen for the calculation of lipase activity.

### 2.7. Calculations and Statistical Design

The following calculations were used for the determination of growth performance, biometry, nutrient digestibility, and biological values, where:

The weight gain (g) was calculated as follows:

  $$\begin{array}{c} \text{Weight gain }\left(g\right)=\text{Final weight }\left(g\right)-\text{initial weight }\left(g\right). \end{array}$$

The weight gain (%) was calculated as follows:

  $$\begin{array}{c} \text{Weight gain }\left(\text{\% }\right)=100\times \left(\frac{\text{final weight }\left(g\right)-\text{initial weight }\left(g\right)}{\text{initial weight }\left(g\right)}\right). \end{array}$$

The specific growth rate (SGR) (% day^−1^) was calculated as follows:

  $$\begin{array}{c} \text{SGR}=100\times \frac{\left(\text{LN final weight}-\text{LN initial weight}\right)}{\text{DOC}}, \end{array}$$

where LN final weight and LN initial weight are the natural log of the final and initial average weight, and the DOC is days of culture period.

The FCR was calculated as follows:

  $$\begin{array}{c} \text{FCR}=\frac{\text{Feed consumed }\left(\text{as fed}\right)}{\text{Weight gain }\left(\text{wet weight}\right)}. \end{array}$$

The daily increment shell length (DISL) (µm day^−1^) was calculated as follows:

  $$\begin{array}{c} \text{DISL }=1000\times \left(\frac{\text{final length}-\text{initial length}}{\text{DOC}}\right), \end{array}$$

where the final length and initial length are measured in millimeters across the longest axis of the shell and DOC is days of culture period.

The feed intake (g abalone^−1^ day^−1^) was calculated as follows:

  $$\begin{array}{c} \text{Feed intake}=\left(\frac{\text{Total feed intake per tank}-\text{estimated uneaten feed}}{\text{Abalone per tank}}\right)/\text{ DOC}, \end{array}$$

where the estimated uneaten feed was assessed by accurately counting the remaining pellets then multiplying by the average weight of a feed pellet on an as-fed basis and the DOC is days of culture period.

The specific feed rate (SFR, %) was calculated as follows:

  $$\begin{array}{c} \text{SFR \% }=\frac{\text{Feed intake}}{{\text{GMW}}^{0.5}}, \end{array}$$

where the GMW is the geometric mean weight of the initial and final average weights.

The retention efficiency (RE, %) of protein and energy were calculated as follows:

  $$\begin{array}{c} \text{RE \% }=100\times \left(\frac{\text{final protein or energy content}-\text{average initial protein or energy content}}{\text{average protein or energy intake}}\right). \end{array}$$

The apparent biological value (ABV) was calculated as follows:

  $$\begin{array}{c} \text{ABV \% }=\frac{\text{Protein or energy RE \% }}{\text{Protein or energy AD}}, \end{array}$$

where AD is apparent digestibility.

The condition factor (Sakai's coefficient) was calculated based on a modified length (mm) × weight (g) relationship using Australian hybrid abalone following methods described by Hayashi [25] as follows:

  $$\begin{array}{c} \text{Condition }\left(\text{Sakai}\right)=\frac{\left(\text{Final weight }\times 1000\right)}{{\text{Final length}}^{3.28}}. \end{array}$$

The calculation of apparent diet nutrient digestibility (AD %) was based on the following formula:

  $$\begin{array}{c} \text{AD \% }=100\times \left[1-\left(\frac{\text{yttrium oxide in diet}}{\text{yttrium oxide faeces}}\right)\times \left(\frac{\text{nutrient in faeces}}{\text{nutrient in diet}}\right)\right]. \end{array}$$

Following previously described methods [16].

For reporting, all data were expressed as mean ± SEM unless otherwise stated. Data were subjected to normality and homogeneity checks before being subjected to a one-way ANOVA in the first experiment and two-way ANOVA in the second experiment. If a significant interaction was observed, then a one-way ANOVA was performed. Levels of significance were defined at *p*  < 0.05 and compared using Tukey's HSD post hoc test. The Pearson's correlation was performed using the Bivariate function, using selected variables and two-tailed significance test (*p*  < 0.05). The IBM SPSS Statistics version 29.0.0.0 (241) was used for all analyses.

## 3. Results

### 3.1. Production Performance

Overall, the abalone adapted to the experimental diets well and achieved over 100% body weight gain in the highest performing diet, with only one abalone death during the experiment. In experiment 1, there were significant differences observed across all the production performance parameters measured, including for final weight, weight gain (g), weight gain %, SGR, FSL, and DISL (Table 4). In all parameters measured, there were no significant differences between the treatments SCP 0%, SCP 5%, or SCP 10%. However, abalone fed diet SCP 20% performed significantly worse than the SCP 0% and SCP 5% only. There was no difference between the SCP 10% and SCP 20% with the exception of the daily shell growth (DISL).

In experiment 2, dietary SCP level (factor A) had a significant effect on all the production parameters measured, including the average final weight, weight gain (g), weight gain %, SGR, FSL, and DISL (Table 4). In all parameters, abalone fed diets without SCP performed significantly better than those fed diets containing 20% SCP. No significant effects of hydrolysate level (factor B) were detected. There were no significant interaction effects recorded between the main factors (A × B).

### 3.2. Feed Retention, Feed Utilization, and Deposition of Protein and Energy Parameters

In experiment 1, there was a significant difference in the feed retention after 16 h, where less recovery (49% ± 5%) was evident in the SCP 5% diet (Table 3). There were no significant differences among any other treatment diets where over 60% was recovered. There was a significant difference in FCR where the SCP 20% diet was higher than the other treatment diets. There was no significant difference in the feed intake up to the SCP 10% level; however, there was a significant difference between the SCP 0% fed abalone and the SCP 20% fed abalone.

In experiment 2 (Table 3), there was a significant difference in the recovered feed after 16 h for both dietary SCP level (factor A) and PH level (factor B), where the SCP 20 diets had higher retention than the SCP 0 diets and the diets without hydrolysate had higher retention than the diets with additional hydrolysate. There was no interaction term noted (A × B). There were significant effects observed for SCP level where both FCR and feed intake were improved by the SCP 0 diets (factor A). While there were no significant differences based on the level of PH. There was no interaction term noted for both FCR and feed intake (A × B). Likewise, there was no significant difference in SFR % for either dietary SCP or hydrolysate level. There were significant differences in the protein retention %, energy retention %, protein ABV %, and the energy ABV % among the treatments, where for each of these parameters, the SCP 0 fed abalone recorded higher values than the SCP 20 fed abalone. There were no significant interaction terms noted for any of the feed utilization parameters examined.

### 3.3. Condition and Soft Body Composition and Analysis of Abalone

In experiment 1, there was a significant difference in the condition factor (Sakai) where abalone fed the SCP 5% diet recording higher values than the other treatments (Table 5). The soft body proportion (%) was significantly lower in the SCP 5% abalone compared to the SCP 0% fed abalone with no difference between SCP 10% and SCP 20%. There were no significant differences between the treatments in any of the proximate composition parameters of soft body tissue.

In experiment 2, there was a significant difference in the abalone condition factor, where SCP 0 recorded the highest values (Table 5). A significant interaction term between dietary SCP and hydrolysate level was observed for the soft body proportion and based on the post hoc pairwise comparison, there was a higher soft body proportion in the SCP 0% + no hydrolysate and SCP 20% + hydrolysate compared to the other treatments. There were no other significant differences in any of the proximate composition parameters of soft body tissue between the dietary treatments.

### 3.4. AD of the Diets and Digestive Enzyme Analysis

In experiment 1, the AD of diet dry matter content was significantly lower in the SCP 5% fed abalone compared to the other treatments (Table 6). This result was consistent among the other macronutrients, where the digestibility of protein, lipid, and carbohydrate was significantly lower in the SCP 5% fed abalone compared to the SCP 20% fed abalone. Accordingly, gross energy digestibility was also lowest in SCP 5%. The diet carbohydrate digestibility was highest in SCP 10% and SCP 20% compared to the other treatments. There were no significant differences in the activities of any of the digestive enzymes analyzed, including *α*-amylase, lipase, or trypsin (Table 6).

In experiment 2, there were significant differences for the AD of dry matter, crude protein, total lipid, carbohydrate, and gross energy with respect to dietary SCP level with the highest digestible values observed in the SCP 20 (Table 6). There was no effect of hydrolysate level nor was there a significant interaction between dietary hydrolysate level and SCP inclusion. Similarly, there were no significant differences in the digestive enzyme analysis of *α*-amylase, lipase, or trypsin for either factor or their interaction (Table 6).

## 4. Discussion

### 4.1. Growth Performance and Feed Utilization

In the present study, the graded inclusion of SCP (PRO-DG, String Bio Pvt. Ltd., Bangalore, India) in the experimental diets fed to Australian hybrid abalone did not significantly affect growth performance up to 10% inclusion, while replacing soy protein concentrate. However, at the highest inclusion of 20%, there was a significant decline in the performance parameters measured. In relative terms, the growth performance at the highest inclusion level of SCP was around 70% of the SCP 0% fed abalone. This drop in weight gain at high inclusion of SCP is consistent with several past studies on fish including yellowtail kingfish [16], Japanese yellowtail [17], Asian seabass [14], Atlantic halibut (*Hippoglossus hippoglossus*) [26], and abalone (*H. discus hannai*) [13]. The overall decline in growth performance observed in these studies may be largely predictable as growth is often simply a reflection of the feed intake response. In this study, this same effect was confirmed to be the case with a decrease in feed intake observed at the highest inclusion level. Changes in growth performance may be attributed to several other factors including the bacterial strain, the chemical composition of the product, how it was manufactured, or on the strategy employed to evaluate it [9, 27]. For example, some studies have shown no differences in growth performance while evaluating SCP. In their study on Pacific white shrimp (*Litopenaeus vannamei*), Chen et al. [28] found a strategy of including a more conservative (yet practical) maximum level of 10.5% SCP (*M. capsulatus*) led to no significant differences in growth parameters. Similarly, in another study on Pacific white shrimp, the inclusion SCP derived from *C. autoethanogenum* did not affect weight gain when included at up to 15% of the diet [29]. In many respects, some experimental outcomes may be simply a result of the experimental design itself (such as using a more conservative approach) as discussed above, or a feature of the specific SCP employed.

Poor palatability of ingredients will likely limit their value to produce acceptable growth; however, in some cases, methods maybe employed to mask or minimise the effect [30]. In both the present experiments, the recorded feed intake declined at the highest inclusion level of SCP. Positively, there were no differences among the other treatments, suggesting that feed intake was not significantly affected up to 10% inclusion. These results are consistent with previous reports examining some bacterial protein meals, indicating a high likelihood of a true palatability effect in Australian hybrid abalone, as seen in other species [13–16]. For example, Wu et al. [13] also reported a decline in feed intake of abalone (*H. discus hannai*) fed increasing SCP (*C. autoethanogenum*) up to 16.25%. In the present study, the addition of a liquid PH did not improve the palatability effect of the high SCP diet. Previously, the production performance and cellular immune function of *H. midae* were improved by the addition of dry PH, but not hydrolyzed fish silage, highlighting clear differences in the efficacy of different hydrolysate products [18]. Therefore, the results of both past and present studies continue to suggest that if the palatability response of certain SCP can be improved then higher inclusion levels of SCP may be achieved. Moreover, the application of specific hydrolyzed protein products requires further validation in abalone species.

Further to this, the results of our first experiment demonstrate the inclusion of SCP at the highest inclusion level led to an increased FCR of the Australian hybrid abalone. Consistent with the reduced feed intake and growth response, these results may suggest that the abalone were not meeting their energetic demands and therefore unable to convert the diet containing high SCP protein as efficiently compared to the soy-based diets. Moreover, bacterial meals are known to contain reasonably high levels of nucleic acids compared to other food stuffs [31, 32]. It has been theorized that some of the reduced performance observed in aquatic feeding studies with incorporating SCP could be attributed to the higher presence of naturally occurring nucleic acids [31]. For example, Ruiz et al. [33] alluded to the impact of nucleic acids on feed intake and growth of rainbow trout juveniles, reporting the optimal inclusion of SCP at only ~42% of fishmeal replacement. Similarly, Aas et al. [34] found that although growth parameters were generally improved without any impact on feed intake in Atlantic salmon (*Salmo salar*), there were significant reductions in digestibility response to increased SCP. In the present study, the total nucleic acid content of the diets was not measured; however, it is reasonable to assume the level would be higher than the soy protein concentrate used, based on the lower ratio of total amino acids to crude protein analyzed (Table 1). Consistent with the present study, a decline in growth performance was observed in Atlantic halibut at the highest inclusion (18%) of SCP tested [26]. Ingested nucleic acids are cleaved into nucleosides by endogenous nuclease enzymes, leading to their conversion to purines and eventual catabolism into uric acid [31, 35, 36]. Consequently, excess uric acid may then become toxic and eventually affect the normal metabolic processes of animals. Therefore, it is plausible that the effects of nucleotides or some unknown antinutrient present in the SCP impacted the downstream metabolism in Australian hybrid abalone, which is consistent with some fish species, affecting the utilization potential.

To date, many of the studies evaluating SCP fed to carnivorous species have included the test ingredient in substitution of fishmeal. This strategy may have led to some of the apparent negative impacts at high inclusion levels on growth performance, noting the importance of fishmeal in the diet of many species. In the present study, we substituted high-quality soy protein concentrate with the novel SCP test ingredient and maintained a constant 5% fishmeal inclusion in all test diets. This strategy was employed for three key reasons, the first being the relative similarity in chemical composition of the SCP and soy protein concentrate used. Second, anecdotally, there is a reluctance to use proteins derived from any land-based rendered animal products in feeds for farmed abalone, hence restricting the available dietary protein sources for this species. Third, fishmeal levels are relatively low in many nutritional investigations on abalone. It remains unclear whether this species has a dietary requirement for fishmeal at all, or what the optimal inclusion level is [12, 13]. Our experimental approach is also consistent with another study investigating the use of SCP up to 20% inclusion and fed to gilthead seabream (*Sparus aurata*). Here, the authors altered plant-based proteins without changing the fishmeal inclusion to balance the diets; however, they reported no differences in the growth performance parameters measured on the gilthead seabream [37].

Additionally, there was a numerical trend toward reduced protein and energy retention at the highest SCP inclusion and this effect was significant in the second experiment. This finding is consistent with the reduced nutrient and amino acid retention reported for halibut fed SCP [26]. In a study on gilthead sea bream juveniles, Marchi et al. [37] reported that the highest inclusions of SCP did lead to a significant reduction in condition factor, protein retention, and plasma urea in the seabream, which may indicate a maximum threshold for inclusion was reached. Together with the present study, these past results are quite convincing that there is a tolerance for maximal replacement of soy protein concentrate with SCP in Australian hybrid abalone diets, as observed in other species. However, several questions arise from this that warrant further explanation, such as the impact of processing conditions on the raw material quality, the impact of palatability and the potential influence of nonprotein nitrogen present in the material.

### 4.2. Pellet Dry Matter Retention

Pellet retention, otherwise referred to as pellet leaching, was examined in the present study to determine if there was a potential impact on any of the growth performance and feed utilization parameters measured. A variety of methods to determine the retention of nutrients in formulated abalone diets have successfully been applied in past studies [11, 38–40]. As mentioned, the Australian hybrid abalone used in the present study display a slow and typically nocturnal feeding behavior and, therefore, it is important to achieve a highly water stable pellet. To address this, we selected ingredients to enhance binding while standardizing the production procedures. In general, our retention results showed high stability after 16 h of soaking in seawater, largely consistent with other studies (e.g., [39]). However, in our study, there was a significant reduction in dry matter retention observed with the SCP 5% diet, compared to the other diets with around 49% of dry matter retained compared to 62%–69% for the other diets. Moreover, these retention results show that the SCP containing diets (experiment 2) were significantly more stable than those formulated with soy protein concentrate, while the addition of PH appeared to reduce their stability. We hypothesize that these differences, although unexpected, likely occurred during the pellet manufacture process; however, this may require further validation.

**Figure 1:**
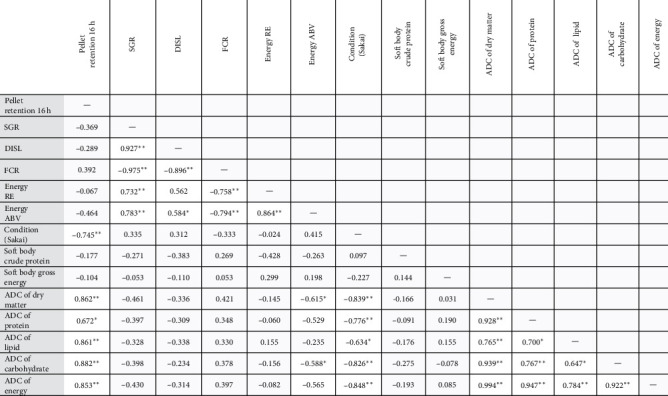
Pearson's correlation matrix of selected variables from experiment 1 (*n* = 12 replicates). ABV, apparent biological value; ADC, apparent digestibility coefficient; DISL, daily increment shell length; FCR, feed conversion ratio; RE, retention; SGR, specific growth rate. *⁣*^*∗*^Correlation is significant at 0.05 level (two-tailed). *⁣*^*∗∗*^Correlation is significant at 0.01 level (two-tailed).

Although the pellet retention result for SCP 5% diet did not lead to a reduction in feed intake or growth performance, it became clear that the pellet retention may have impacted the overall condition factor of abalone fed that treatment. Importantly, several of the measured parameters were significantly correlated to the retention values of the feeds (Figure 1). Moreover, the AD of the diet nutrients was significantly correlated to the pellet retention, and it did not follow a dose:response relationship consistent with the other treatments in the array. This significant correlation was primarily driven by the SCP 5% diet retention values. There was, however, no correlation observed between the production performance parameters and pellet retention. Moreover, a reduction in growth performance [41] and digestibility [42] associated with pellet retention was previously demonstrated. Clearly, the apparent disparity between pellet retention and some measured parameters presented here warrants further investigation; however, given no negative association between pellet retention and growth performance, it may be most relevant to explore this within the context of the physical properties of diets.

### 4.3. AD of Diets

An important part of any new or novel ingredient characterization process is understanding its AD value [30, 43]. In this study, we measured the diet digestibility of dry matter and the key macronutrients. Although this technique does not consider the specific effect of the ingredient in isolation, there is a reduced potential for the amplification of calculation errors compared to individual ingredient digestibility [44]. As previously mentioned, the SCP 5% diet may have been affected by the pellet manufacture process (or another unidentified feature); therefore, when considering the other diets in experiment 1, dry matter digestibility values were not different from each other. In general, the dry matter digestibility was acceptable (~57%–61%) and confirmed by the addition of digestible protein, digestible lipid, and digestible carbohydrate. The range of dry matter digestibility is consistent with values previously reported for subadult Australian hybrid abalone under similar experimental conditions (~68%–70%) [45] while lower than the value previously reported for (~87%) for *H. midae* using a high fishmeal diet [46]. The present data are also consistent with smaller *H. laevigata* where values of around ~73% have been reported when held at a similar water temperature [47]. It should be noted that in the latter study, it was also found that the older abalone (3 vs. 2 years) demonstrated a significantly higher AD of dry matter and crude protein, which may in part explain the slight differences observed in our study using 1-year-old stock.

In experiment 1, the AD of macronutrients and energy were unaffected by SCP inclusion level, withstanding the already mentioned effect of the SCP 5% diet retention. However, examining the results of experiment 2, there is a clear improvement in AD of all dietary macronutrients in the SCP 20 treatment abalone compared to those in the SCP 0 treatment. This positive effect is based on the ingredient and, the addition of the PH product had no impact on the diet digestibility values. Naturally, it is unclear whether the increased digestibility of crude protein in SCP 20 is due to the elevated SCP level or the concomitant reduction of soy protein concentrate. This result highlights the importance of fully characterizing raw materials, despite the potential for increasing error through calculation, as some discrete differences may be very positive and beneficial. For example, in a study using different iterations of the same *M. capsulatus* product fed to Pacific white shrimp, compared to both fishmeal and soy protein concentrate, there were no significant differences in crude protein or energy digestibility of the ingredients. However, the digestibility of the sum of amino acids and certain individual amino acids were significantly different, among the SCP products compared to the fishmeal examined [48]. Similarly, Salze and Tibbetts [49] reported the AD of protein and energy from *Methylobacterium extorquens* in presmolt Atlantic salmon to be ~85% and ~78%, respectively, which is indicative of a highly digestible product, comparable to high-quality fishmeal for Atlantic salmon. Therefore, establishing species-specific values of ingredient digestibility certainly warrants further investigation, as data are lacking in Australian hybrid abalone. These data may allow the formulation of future feeds to be informed by reliable digestibility data across the suite of raw materials commonly used.

### 4.4. Digestive Enzyme Activity

Digestive enzyme activity is often measured in abalone nutrition studies, with *α*-amylase, trypsin, and lipase frequently targeted [12, 13, 50–53]. The digestive enzyme activity will depend on several factors such as substrate contact time [47], diet type [50, 54], and temperature [51]. In both the experiments herein, there were no significant differences in activity for any of the targeted digestive enzymes. In a recent study by Bansemer et al. [51], the digestive enzyme activity was measured in the gastrointestinal tract of postweaned (~2 g final weight); *H. laevigata* and the activity of *α*-amylase and lipase were higher than in the present study for larger Australian hybrid abalone. However, trypsin activity was low across the two studies. This potentially confirms that the trypsin activity in abalone is functionally low and is primarily modulated by water temperature rather than diet changes. Moreover, *α*-amylase was shown to positively respond to increasing protein with a concomitant reduction in dietary starch in greenlip [50] and pacific abalone [54]; however, beyond the optimal level of protein, the activity was reduced. Ma et al. [55] found higher *α*-amylase activity in the digestive gland of abalone (*H. discuss hannai*) when fed higher levels of kelp meal and Liu et al. [56] found that there was a general increasing trend of enzyme activity with increased dietary docosahexaenoic acid. The enzyme activity results across studies are difficult to compare due to large variation in the values observed; however, it could be argued that with increasing growth, there is a higher expression of digestive enzymes. In the present study, the diets were iso-proteic with the main differences being the raw materials selected and therefore this same effect on digestive enzyme activity was not observed. However, the fact that SCP did not significantly affect the enzyme activity, while the growth response was reduced, it could be argued that the activity was indeed relatively higher.

## 5. Conclusion

The outcomes of this study have demonstrated that it is feasible to include SCP derived from *M. capsulatus* up to 10% in Australian hybrid abalone diet formulations while substituting soy protein concentrate. This study shows that SCP inclusion beyond this level may be problematic and lead to a reduction in production performance. A notable positive influence of the SCP on diet digestibility was observed and this result combined with the altered growth performance hints at a more metabolic issue rather than one of simply feed intake when assessing levels up to 20% in the diet of Australian hybrid abalone.

## Figures and Tables

**Table 1:** Key ingredient composition presented on dry matter basis (g kg^−1^) unless otherwise shown.

Nutrient composition (g kg^−1^)	Soy protein concentrate	PRO-DG (SCP)
Moisture (g kg^−1^ as is basis)	70.3	63.4
Crude protein (*N* × 6.25)	639.5	739.3
Total lipid	12.8	113.4
Ash	72.4	76.7
Carbohydrates^a^	275.3	70.6
Gross energy (MJ kg^−1^ DM)^b^	20.3	23.1
Amino acids (g kg^−1^ DM)		
Alanine	27.4	48.9
Arginine	41.9	43.6
Aspartic acid	38.3	29.4
Glutamic acid	117.6	75.0
Glycine	25.1	33.4
Histidine	14.4	13.2
Isoleucine	34.0	36.9
Leucine	48.0	52.3
Lysine	28.6	27.4
Methionine	7.4	19.8
Phenylalanine	21.9	20.4
Proline	37.4	30.8
Serine	32.9	24.1
Threonine	24.9	30.9
Tyrosine	17.9	21.7
Valine	26.8	36.5
Total	544.6	544.2
Ratio *N* × 6.25 to total (%)	85.2	73.6

Abbreviation: SCP, single cell protein.

^a^Carbohydrates calculated (1000 − sum [ash + protein + lipid]).

^b^Gross energy calculated based on standard factors of protein, lipid, and carbohydrate values.

**Table 2:** Diet formulation and composition for both experiments 1 and 2.

Ingredients (%)	SCP 0%	SCP 5%	SCP 10%	SCP 20%	SCP 0% + PH	SCP 20% + PH
Fishmeal (anchovy)^a^	5.00	5.00	5.00	5.00	5.00	5.00
Soy protein concentrate^a^	26.00	20.00	15.00	4.00	26.00	4.00
PRO-DG (SCP)^b^	0.00	5.00	10.00	20.00	0.00	20.00
Plant-based ingredients^c^	63.83	65.03	65.68	67.38	64.10	66.98
Micro additions^d^	2.01	1.91	1.86	1.76	1.76	2.01
Mono calcium phosphate	2.10	2.00	1.50	1.00	2.10	1.00
Yttrium oxide	0.05	0.05	0.05	0.05	0.05	0.05
Canola oil:fish oil (50:50)	1.00	1.00	1.00	1.00	1.00	1.00
Protein hydrolysate (PH)^e^	—	—	—	—	3.00	3.00
Chemical composition (g kg^−1^)						
Moisture	76.0	47.8	48.2	55.4	55.3	54.8
Crude protein	432.4	427.3	429.5	432.7	437.2	439.8
Total lipid	34.8	34.6	34.7	43.0	44.7	28.2
Ash	55.5	56.0	50.9	50.5	49.8	57.0
Carbohydrates^f^	477.3	482.0	485.0	473.9	468.3	475.1
Gross energy (MJ kg^−1^ DM)^g^	19.8	19.7	19.8	20.1	20.1	19.7
Amino acids (g kg^−1^ DM)						
Arginine	24.1	24.4	22.9	22.6	20.6	22.6
Histidine	8.9	9.2	8.7	8.8	7.7	8.6
Isoleucine	22.7	22.4	21.3	21.1	19.3	20.2
Leucine	30.7	30.4	29.5	29.7	27.5	28.9
Lysine	14.1	13.6	12.8	13.4	12.4	13.6
Methionine	8.8	9.1	9.4	10.2	7.9	8.6
Phenylalanine	14.2	13.8	13.3	13.0	13.3	12.1
Threonine	14.4	14.4	14.3	14.7	13.7	13.7
Valine	18.5	18.4	18.4	18.9	17.0	16.6

*Note:* Ingredients are formulated as percentage inclusion. Chemical composition data are presented on a dry matter basis (g kg^−1^).

Abbreviation: SCP, single cell protein.

^a^Ridley Agriproducts Pty Ltd., Narangba, Queensland, Australia.

^b^SCP; single cell protein, String Bio Pvt. Ltd., Bangalore, India.

^c^Plant-based ingredients include wheat flour and pregelled wheat starch from Manildra Group, Altona North, Victoria, Australia, wheat gluten from Ridley Agriproducts Pty Ltd., Narangba, Queensland, Australia, and lupin kernel meal from Coorow Seeds, Western Australia, Australia.

^d^Includes vitamin and mineral premix (proprietary Ridley Agriproducts Pty Ltd.), astaxanthin, soy lecithin, choline chloride 70%, vitamin C, vitamin E, methionine, and lysine.

^e^Fish Trade International Pty Ltd., Western Australia, Australia.

^f^Carbohydrates calculated (1000 − sum [ash + protein + lipid]).

^g^Gross energy calculated based on standard factors of protein, lipid, and carbohydrate values.

**Table 3:** Feed retention testing (leaching), feed utilization, and deposition values of protein and energy from experiments 1 and 2.

Treatment effects	Retention (%) (16 h)	FCR	FI (g abalone^−1^ day^−1^)	SFR (%)	Protein RE (%)	Energy RE (%)	Protein ABV (%)	Energy ABV (%)
Experiment 1								
Treatment means								
SCP 0%	62.33 ± 2.08^a^	0.87 ± 0.02^a^	0.1094 ± 0.00^a^	0.74 ± 0.01	45.91 ± 3.31	31.19 ± 2.45	68.3 ± 7.27	50.42 ± 5.55
SCP 5%	49.33 ± 4.93^b^	0.93 ± 0.09^a^	0.1052 ± 0.00^ab^	0.73 ± 0.02	43.99 ± 9.32	29.13 ± 7.00	74.12 ± 16.59	54.60 ± 12.73
SCP 10%	63.33 ± 2.89^a^	0.95 ± 0.06^a^	0.1010 ± 0.01^ab^	0.71 ± 0.01	42.71 ± 3.82	28.36 ± 2.21	62.79 ± 5.34	44.55 ± 3.15
SCP 20%	69.00 ± 1.00^a^	1.12 ± 0.07^b^	0.0984 ± 0.00^b^	0.73 ± 0.01	39.85 ± 2.90	26.45 ± 3.49	56.84 ± 4.00	40.18 ± 4.51
One-way ANOVA	0.001	0.007	0.024	0.208	0.609	0.610	0.231	0.169
Experiment 2								
SCP level (A)								
SCP 0	58.67 ± 4.23	0.92 ± 0.08	0.1080 ± 0.00	0.74 ± 0.01	46.45 ± 3.10	31.02 ± 2.44	69.73 ± 6.28	51.45 ± 4.64
SCP 20	67.33 ± 2.80	1.11 ± 1.11	0.9960 ± 0.00	0.73 ± 0.73	37.33 ± 37.33	24.83 ± 24.83	52.30 ± 52.30	37.23 ± 37.23
Protein hydrolysate level (B)								
PH 0	65.67 ± 3.93	1.00 ± 0.14	0.1039 ± 0.01	0.74 ± 0.01	42.88 ± 4.33	28.82 ± 3.75	62.57 ± 8.18	45.30 ± 7.20
PH 3	60.33 ± 6.19	1.03 ± 0.10	0.1037 ± 0.00	0.74 ± 0.01	40.90 ± 7.56	27.02 ± 5.15	59.46 ± 14.14	43.38 ± 11.18
Two-way ANOVA								
A	0.001	0.002	<0.001	0.153	0.002	0.010	0.001	0.002
B	0.002	0.509	0.846	0.760	0.368	0.357	0.417	0.551
A × B	0.118	0.151	0.072	0.760	0.178	0.453	0.139	0.235
Treatment means								
SCP 0% + PH 0	62.33 ± 2.08	0.87 ± 0.02	0.1094 ± 0.00	0.74 ± 0.01	45.91 ± 3.31	31.19 ± 2.45	68.30 ± 7.27	50.42 ± 5.55
SCP 20% + PH 0	69.00 ± 1.00	1.12 ± 0.07	0.0984 ± 0.00	0.73 ± 0.01	39.85 ± 2.90	26.45 ± 3.49	56.84 ± 4.00	40.18 ± 4.51
SCP 0% + PH 3	55.00 ± 0.00	0.97 ± 0.10	0.1066 ± 0.00	0.74 ± 0.00	46.98 ± 3.49	30.84 ± 2.97	71.16 ± 6.30	52.47 ± 4.45
SCP 20% + PH 3	65.67 ± 3.21	1.09 ± 0.08	0.1008 ± 0.00	0.73 ± 0.01	34.82 ± 4.46	23.20 ± 3.70	47.76 ± 7.06	34.28 ± 6.65

*Note:* Data are presented as mean ± SEM. Experiment 1 treatments are *n* = 3 replicates while experiment 2 treatments are *n* = 6 replicates for each factor. Values followed by different superscript letters in the same column indicate significant differences (Tukey's HSD; *p*  < 0.05).

Abbreviations: ABV, apparent biological value; FCR, feed conversion ratio; FI, feed intake; PH, protein hydrolysate; RE, retention; SCP, single cell protein; SFR, specific feed rate.

**Table 4:** Growth performance of abalone experiments 1 and 2.

Treatment effects	Initial weight (g)	Average weight (g)	Weight gain (g)	Weight gain (%)	SGR (% day^−1^)	ISL (mm)	FSL (mm)	DISL (*µ*m abalone^−1^ day^−1^)
Experiment 1								
Treatment means								
SCP 0%	9.25 ± 0.25	21.03 ± 0.09^a^	11.77 ± 0.22^a^	127.30 ± 5.56^a^	0.87 ± 0.03^a^	41.72 ± 0.21	53.59 ± 0.18^a^	126.26 ± 2.51^a^
SCP 5%	9.27 ± 0.16	19.98 ± 0.91^a^	10.72 ± 1.00^a^	115.76 ± 11.98^a^	0.82 ± 0.06^a^	41.39 ± 0.41	52.34 ± 0.70^ab^	116.49 ± 4.20^a^
SCP 10%	9.25 ± 0.20	19.28 ± 1.20^ab^	10.03 ± 1.05^ab^	108.38 ± 9.74^ab^	0.78 ± 0.05^ab^	41.36 ± 0.32	52.24 ± 0.88^ab^	115.72 ± 6.86^a^
SCP 20%	9.26 ± 0.10	17.53 ± 0.72^b^	8.27 ± 0.70^b^	89.33 ± 7.54^b^	0.68 ± 0.05^b^	41.33 ± 0.73	50.80 ± 0.65^b^	100.72 ± 6.77^b^
* *One-way ANOVA	1	0.006	0.005	0.005	0.005	0.71	0.006	0.003
Experiment 2								
SCP level (A)								
SCP 0	9.27 ± 0.24	20.39 ± 0.96	11.12 ± 1.06	120.03 ± 12.96	0.84 ± 0.07	41.6 ± 0.23	52.98 ± 0.97	121.04 ± 8.38
SCP 20	9.25 ± 9.25	17.77 ± 17.77	8.51 ± 8.51	91.99 ± 91.99	0.69 ± 0.69	41.43 ± 41.43	51.13 ± 51.13	103.23 ± 103.23
Protein hydrolysate level (B)								
PH 0	9.26 ± 0.17	19.28 ± 1.97	10.02 ± 1.97	108.31 ± 21.63	0.78 ± 0.11	41.53 ± 0.53	52.20 ± 1.59	113.49 ± 14.71
PH 3	9.27 ± 0.19	18.88 ± 1.26	9.61 ± 1.28	103.71 ± 14.42	0.75 ± 0.08	41.50 ± 0.16	51.91 ± 0.95	110.78 ± 9.55
Two-way ANOVA								
A	0.857	0.001	0.001	0.001	0.001	0.474	0.002	0.003
B	0.900	0.380	0.384	0.426	0.483	0.889	0.508	0.530
A × B	0.835	0.076	0.081	0.108	0.117	0.376	0.054	0.098
Treatment means								
SCP 0% + PH 0	9.25 ± 0.25	21.03 ± 0.09	11.77 ± 0.22	127.3 ± 5.56	0.87 ± 0.03	41.72 ± 0.21	53.59 ± 0.18	126.26 ± 2.51
SCP 20% + PH 0	9.26 ± 0.10	17.53 ± 0.72	8.27 ± 0.70	89.33 ± 7.54	0.68 ± 0.05	41.33 ± 0.73	50.80 ± 0.65	100.72 ± 6.77
SCP 0% + PH 3	9.29 ± 0.28	19.75 ± 1.05	10.46 ± 1.20	112.76 ± 15.17	0.80 ± 0.08	41.47 ± 0.21	52.36 ± 1.09	115.82 ± 9.37
SCP 20% + PH 3	9.25 ± 0.11	18.01 ± 0.76	8.76 ± 0.68	94.66 ± 6.61	0.71 ± 0.04	41.52 ± 0.13	51.45 ± 0.66	105.74 ± 7.99

*Note:* Data are presented as mean ± SEM. Experiment 1 treatments are *n* = 3 replicates while experiment 2 treatments are *n* = 6 replicates for each factor. Values followed by different superscript letters in the same column indicate significant differences (Tukey's HSD; *p*  < 0.05).

Abbreviations: DISL, daily increment shell length; FCR, feed conversion ratio; FI, feed intake; FSL, final shell length; ISL, initial shell length; PH, protein hydrolysate; SCP, single cell protein; SFR, specific feed ratio; SGR, specific growth rate.

**Table 5:** Condition and soft body proportion and analysis of abalone (soft body) from experiments 1 and 2.

Treatment effects	Condition (Sakai)	Soft body proportion (%)	Moisture (%)	Crude protein (%)	Total lipid (%)	Ash (%)	Carbohydrate (%)	Gross energy (MJ kg^−1^ DM)
Experiment 1								
Treatment means								
SCP 0%	85.90 ± 1.0^b^	64.91 ± 0.23^a^	75.98 ± 0.95	66.88 ± 1.27	7.33 ± 0.21	10.88 ± 0.9	14.90 ± 1.38	21.25 ± 0.17
SCP 5%	88.18 ± 0.61^a^	61.17 ± 1.78^b^	76.46 ± 2.18	67.75 ± 1.00	7.07 ± 0.14	10.37 ± 0.86	14.81 ± 1.47	21.33 ± 0.12
SCP 10%	85.54 ± 0.86^b^	62.54 ± 0.51^ab^	76.63 ± 0.56	67.31 ± 1.20	7.04 ± 0.24	10.46 ± 0.65	15.20 ± 1.01	21.28 ± 0.20
SCP 20%	85.27 ± 0.09^b^	61.55 ± 0.25^b^	75.41 ± 0.91	67.36 ± 2.15	7.37 ± 0.74	10.65 ± 0.86	14.62 ± 1.91	21.32 ± 0.19
One-way ANOVA	0.004	0.006	0.677	0.909	0.668	0.873	0.968	0.928
Experiment 2								
SCP level (A)								
SCP 0	86.47 ± 1.21	63.12 ± 2.01	75.91 ± 1.09	68.40 ± 2.10	7.20 ± 0.38	10.64 ± 0.81	13.76 ± 1.60	21.36 ± 0.21
SCP 20	84.65 ± 84.65	62.86 ± 62.86	75.77 ± 75.77	66.93 ± 66.93	7.52 ± 7.52	11.41 ± 11.41	14.15 ± 14.15	21.2 ± 21.20
Protein hydrolysate level (B)								
PH 0	85.59 ± 0.72	63.23 ± 1.85	75.7 ± 0.89	67.12 ± 1.60	7.35 ± 0.49	10.77 ± 0.80	14.76 ± 1.50	21.29 ± 0.17
PH 3	85.53 ± 2.06	62.75 ± 1.88	75.98 ± 1.29	68.21 ± 2.18	7.37 ± 0.55	11.28 ± 1.20	13.14 ± 1.01	21.27 ± 0.26
Two-way ANOVA								
A	0.020	0.615	0.846	0.134	0.333	0.149	0.627	0.152
B	0.929	0.360	0.695	0.250	0.962	0.319	0.071	0.859
A × B	0.096	0.001	0.551	0.057	0.390	0.071	0.410	0.047
Treatment means								
SCP 0% + PH 0	85.90 ± 1.00	64.91 ± 0.23^a^	75.98 ± 0.95	66.88 ± 1.27	7.33 ± 0.21	10.88 ± 0.90	14.90 ± 1.38	21.25 ± 0.17
SCP 20% + PH 0	85.27 ± 0.09	61.55 ± 0.25^b^	75.41 ± 0.91	67.36 ± 2.15	7.37 ± 0.74	10.65 ± 0.86	14.62 ± 1.91	21.32 ± 0.19
SCP 0% + PH 3	87.03 ± 1.31	61.34 ± 0.67^b^	75.83 ± 1.43	69.93 ± 1.56	7.07 ± 0.52	10.39 ± 0.82	12.61 ± 0.73	21.46 ± 0.20
SCP 20% + PH 3	84.02 ± 1.44	64.16 ± 1.56^a^	76.13 ± 1.43	66.50 ± 0.80	7.66 ± 0.48	12.16 ± 0.76	13.68 ± 1.08	21.07 ± 0.12

*Note:* Soft body proximate compositional data are presented on a dry matter basis. Data are presented as mean ± SEM. Experiment 1 treatments are *n* = 3 replicates while experiment 2 treatments are *n* = 6 replicates for each factor. Values followed by different superscript letters in the same column indicate significant differences (Tukey's HSD; *p*  < 0.05).

Abbreviations: PH, protein hydrolysate; SCP, single cell protein.

**Table 6:** Apparent digestibility of diets and digestive enzyme activity from experiments 1 and 2.

Treatment effects	Dry matter (%)	Protein (%)	Lipid (%)	Carbohydrate (%)	Energy (%)	*α*-Amylase (U mg^−1^ protein)	Lipase (U mg^−1^ protein)	Trypsin (U mg^−1^ protein)
Experiment 1								
Treatment means								
SCP 0%	57.14 ± 2.53^a^	67.42 ± 3.62^ab^	63.68 ± 6.07^ab^	55.02 ± 1.93^b^	62.02 ± 2.14^a^	16.99 ± 3.73	0.22 ± 0.04	0.87 ± 0.15
SCP 5%	48.49 ± 1.81^b^	59.53 ± 5.04^b^	51.53 ± 8.92^b^	45.98 ± 0.22^c^	53.29 ± 2.48^b^	17.75 ± 1.76	0.18 ± 0.05	0.87 ± 0.45
SCP 10%	59.86 ± 1.32^a^	68.04 ± 2.9^ab^	61.34 ± 6.12^ab^	58.68 ± 0.97^a^	63.64 ± 1.40^a^	16.41 ± 3.83	0.16 ± 0.07	0.66 ± 0.09
SCP 20%	61.30 ± 2.27^a^	70.14 ± 3.15^a^	73.47 ± 5.16^a^	58.61 ± 1.60^a^	65.74 ± 2.65^a^	13.74 ± 3.72	0.16 ± 0.06	0.85 ± 0.25
One-way ANOVA	0.001	0.038	0.025	0.001	0.001	0.526	0.583	0.745
Experiment 2								
SCP level (A)								
SCP 0	55.59 ± 3.43	66.76 ± 2.82	58.12 ± 7.91	52.71 ± 4.47	60.41 ± 3.10	15.95 ± 2.70	0.21 ± 0.04	0.82 ± 0.14
SCP 20	62.42 ± 62.42	71.59 ± 71.59	74.14 ± 74.14	59.29 ± 59.29	66.85 ± 66.85	15.11 ± 15.11	0.19 ± 0.19	1.28 ± 1.28
Protein hydrolysate level (B)								
PH 0	59.22 ± 3.13	68.78 ± 3.38	68.58 ± 7.36	56.82 ± 2.53	63.88 ± 2.96	15.36 ± 3.78	0.19 ± 0.06	0.86 ± 0.19
PH 3	58.79 ± 6.07	69.57 ± 4.24	63.69 ± 12.69	55.18 ± 6.90	63.39 ± 5.71	15.70 ± 1.51	0.20 ± 0.06	1.25 ± 0.88
Two-way ANOVA								
A	0.004	0.018	0.001	0.016	0.004	0.620	0.542	0.218
B	0.808	0.641	0.120	0.473	0.765	0.841	0.703	0.291
A × B	0.163	0.232	0.057	0.207	0.126	0.174	0.382	0.205
Treatment means								
SCP 0% + PH 0	57.14 ± 2.53	67.42 ± 3.62	63.68 ± 6.07	55.02 ± 1.93	62.02 ± 2.14	16.99 ± 3.73	0.22 ± 0.04	0.87 ± 0.15
SCP 20% + PH 0	61.30 ± 2.27	70.14 ± 3.15	73.47 ± 5.16	58.61 ± 1.60	65.74 ± 2.65	13.74 ± 3.72	0.16 ± 0.06	0.85 ± 0.25
SCP 0% + PH 3	54.04 ± 3.97	66.10 ± 2.35	52.56 ± 5.20	50.39 ± 5.49	58.81 ± 3.43	14.91 ± 1.04	0.20 ± 0.05	0.78 ± 0.15
SCP 20% + PH 3	63.53 ± 2.97	73.04 ± 1.85	74.81 ± 2.01	59.96 ± 4.50	67.96 ± 2.62	16.49 ± 1.65	0.21 ± 0.09	1.71 ± 1.13

*Note:* Data are presented as mean ± SEM. Experiment 1 treatments are *n* = 3 replicates while experiment 2 treatments are *n* = 6 replicates for each factor. Values followed by different superscript letters in the same column indicate significant differences (Tukey's HSD; *p*  < 0.05).

Abbreviations: PH, protein hydrolysate; SCP, single cell protein.

## Data Availability

The data that support the findings of this study are available from the corresponding author upon reasonable request.
